# Investigating the spatial micro-epidemiology of diseases within a point-prevalence sample: a field applicable method for rapid mapping of households using low-cost GPS-dataloggers

**DOI:** 10.1016/j.trstmh.2011.05.007

**Published:** 2011-09

**Authors:** J. Russell Stothard, Jose C. Sousa-Figueiredo, Martha Betson, Edmund Y.W. Seto, Narcis B. Kabatereine

**Affiliations:** a CTID, Liverpool School of Tropical Medicine, Pembroke Place, Liverpool L3 5QA, UK; b Department of Infectious and Tropical Diseases, London School of Hygiene & Tropical Medicine, Keppel Street, London, WC1E 7HT, UK; c School of Public Health, University of California, Berkeley, California, USA; d Vector Control Division, Ministry of Health, Kampala, P.O. 1551, Uganda

**Keywords:** Intestinal schistosomiasis, Malaria, Hookworm, Co-infection, I-GotU, Crowdsourcing

## Abstract

Point-prevalence recording of the distribution of tropical parasitic diseases at village level is usually sufficient for general monitoring and surveillance. Whilst within-village spatial patterning of diseases exists, and can be important, mapping infected cases in a household-by-household setting is arduous and time consuming. With the development of low-cost GPS-data loggers (< £40) and available GoogleEarth^TM^ satellite imagery, we present a field-applicable method based on crowdsourcing for rapid identification of infected cases (intestinal schistosomiasis, malaria and hookworm) by household. A total of 126 mothers with their 247 preschool children from Bukoba village (Mayuge District, Uganda) were examined with half of these mothers given a GPS-data logger to walk home with, returning the unit the same day for data off-loading, after which, households were assigned GPS coordinates. A satellite image of Bukoba was annotated with households denoting the infection status of each mother and child. General prevalence of intestinal schistosomiasis, malaria and hookworm in mothers and children was: 27.2 vs 7.7%, 28.6 vs 87.0% and 60.0 vs 22.3%, respectively. Different spatial patterns of disease could be identified likely representing the intrinsic differences in parasite biology and interplay with human behaviour(s) across this local landscape providing a better insight into reasons for disease micro-patterning.

## 1 Introduction

The distribution of many tropical parasitic diseases is a complex interplay of parasite biology (as well as associated vectors or intermediate hosts thereof), suitability of the surrounding local environment and human-related factors, such as our biology and physiology, demography, and behaviour.^1^, ^2^ Where this complex interplay is permissive it gives rise to a disease-endemic landscape, and where it is not delineates its boundaries or absence. Such patterns can be temporal and operate at different scales, from the macro to the micro, the causal factors for which may or may not transfer across scales.^2^, ^3^, ^4^ For example, at the macro level, areas may simply be too hot or cold to sustain parasite transmission and whilst these thermal boundaries may still apply at the micro level, others become more influential, such as the numbers of infected people needed to sustain sufficient parasites in local transmission.^5^ Thus, at this fine scale level, parasites must exceed certain population thresholds to pass successfully from humans to their vectors/intermediate hosts, and vice versa, or sufficiently contaminate the environment as in the case of soil-transmitted helminths, to safeguard their infection potential(s).^6^, ^7^

Assessing the transmission potential or actual patterns of endemicity at the micro-level is particularly challenging as a variety of potentially unique place-specific factors are involved; foremost, a detailed cartographical knowledge of the local area is needed which can be logistically challenging to record, especially if this knowledge is held verbally alone, i.e. distribution of households within a village.^8^ In the African rural setting, households rarely have a numerical ordering system, the absence of which hinders a quick appreciation of where people may originate, or be drawn from during a disease point-prevalence spot check. This is often the case for mapping of schistosomiasis, malaria and soil-transmitted helminthiasis surveys (see information reported in www.thiswormyworld.org for helminths and www.map.ox.ac.uk for malaria), where the cartographical level below the level of village, typically of 4–5 km^2^ area, is not generally investigated.^5^, ^9^, ^10^, ^11^, ^12^ Nonetheless efforts to collect this fine-scale information have been rewarded by a deeper understanding of general disease epidemiology, especially the concept of polyparasitism, dynamics of individual host morbidity and local environmental risk.^13^, ^14^, ^15^, ^16^ Better knowledge of households’ location, and navigating the small footpaths to find them, also plays an assisting role in better community mobilization in longitudinal studies, but at the same time raises issues over privacy and participation.^17^

Developing field applicable methods to map, more rapidly, the location of households is therefore very much needed.^18^, ^19^ Despite ongoing advances in handheld global positioning system (GPS) technology, it is only recently that units have become affordable for more widespread application(s) as this technology has become mainstream and, in so doing, lowered in price.^20^ Two other contingent factors are also relevant. Firstly, the units themselves have undergone progressive miniaturization and taken on board data logging capacities, able to store several thousand positional coordinates.^21^ Secondly, these units can interface with laptop computers running ‘free’ geographical information system (GIS) software such as GoogleEarth^TM^, which has allowed easy plotting and overlaying of recorded locations onto base maps/high resolution satellite images as never before. Such developments have allowed for a new method in the geospatial sciences known as ‘GPS crowdsourcing’ in which spatial phenomena, e.g., presence of people, roads, and traffic, are inferred from continual GPS measurements.^22^

Here, we conduct a point-prevalence study undertaken in mothers and their preschool children in a typical Ugandan village on the shoreline of Lake Victoria. Using handheld GPS-data logging units, we investigate the within-village disease patterning, or spatial micro-epidemiology, of intestinal schistosomiasis, malaria and hookworm.

## 2 Methods

### 2.1 Study area

**Figure 1:**
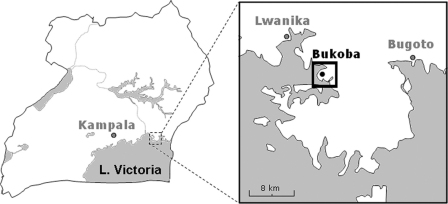
Schematic map of Uganda with part of the Mayuge District magnified: Bukoba village is situated on the Mayuge peninsula on the Lake Victoria shoreline. Lwanika and Bugoto were the other two villages surveyed in the large-scale parasitological survey.

The study was conducted in June 2009 in the lakeshore village of Bukoba (0.311061°N, 33.49240°E), Mayuge District, Uganda on the northern shoreline of Lake Victoria, see Figure 1. This village is one of three selected in Mayuge District where a cohort of mothers and preschool children has been recruited into a longitudinal monitoring study. In this cohort, the infection dynamics of intestinal schistosomiasis, malaria and soil-transmitted helminthiasis are being studied in the face of regular de-worming and home-based management of malaria.

**Figure 2:**
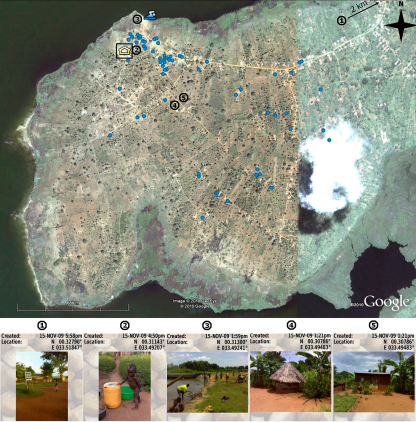
GoogleEarth^TM^ map of Bukoba village with households annotated as blue circles. Other annotations include the blue swimmer identifying the primary water-contact site for the village as well as the yellow house, identifying the church which served as team headquarters during the study. The images were captured and ‘geostamped’ using the Oregon 550t unit: (1) the closest heath care centre (HC IV), around 2 km outside the map; (2) a small child about to be bathed in a basin full of water collected from the lakeshore; (3) the primary water-contact site for the village, with villagers’ normal water-contact behaviour (washing bicycles or clothes, collecting water or getting on boat); (4) a thatched hut with mud walls and (5) a tin-roofed house with wall made from brick.

Bukoba is spread across an area of approximately 3.5 km^2^ with homesteads more aggregated along the northern side following a linear development along the Mayuge Town road which terminates at the lakeshore where construction sand is harvested, see Figure 2. The western-southern perimeter of the village borders on Lake Victoria although access to the lake is very restricted being blocked by thick papyrus reed beds.

Although the precise number of inhabitants in Bukoba is not accurately known as registers are poorly kept, it is in the region of 2000 people, locally serviced by several shops, a primary school and church. From interviews with cohort members, fishing is the occupation of a small minority of mothers (<2%) despite Bukoba being located on the lake, while the vast majority of mothers (94%) are occupied in subsistence farming on small holdings, and cash crop production such as tomatoes, maize and cassava. As there is no borehole in Bukoba, household water is drawn daily, directly from the lake at various collection points, mainly from the northern shoreline. General sanitation and hygiene is reasonable with nearly all having communal access to deep shaft pit latrines. On-site electricity is provided by portable generator or batteries alone.

### 2.2 Mother and child cohort and parasitological screening

After conducting village sensitization in June 2009 making use of the village chairman, village council and associated community drug distributors for community mobilization, the study objectives were explained to all attending mothers (thought to be about 80% of the eligible population). After obtaining verbal consent, a mother and child cohort consisting of 126 mothers (mean age 29 years, range 17–45 years) with 247 preschool children (mean age 3 years, range 0.5–6 years, 51% male) was selected for subsequent monitoring. Written informed consent was obtained upon interview (formal recruitment) either as a signature or thumbprint (53% were illiterate) where a suite of verbal questions were also asked pertaining to socio-economic status, putative risk factors for intestinal schistosomiasis, malaria and soil-transmitted helminthiasis, as well as access to preventive measures e.g. bednets and medication such as anthelminthics.

During a working week, each participant submitted two consecutive-day stool samples for examination of *Schistosoma mansoni* eggs and ova from soil-transmitted helminths. From each stool two Kato-Katz thick smears (2 x 41.7 mg) on the same slide were made. Slides were then inspected under the light microscope at x100 magnification and infections were classified according to established WHO categories for all encountered helminths. Fingerprick blood was taken using a disposable safety lancet to prepare a thick and thin Giemsa-stained blood film and to conduct a Paracheck^©^ rapid diagnostic test (Orchid Plc, Goa, India). Blood films were inspected on site for occurrence of *Plasmodium* spp. by light microscopy at x1000 under oil immersion. The results of each test were tallied and entered electronically using EpiData^TM^ 3.1 software (The EpiData Association, Odense, Denmark) and analysis was then performed with R 2.10.1 statistical package^®^ (The R Foundation for Statistical Computing, Vienna, Austria) to obtain general prevalence and 95% confidence intervals estimated.

After parasitological examination, regardless of infection status, all persons were treated with a standard dose of praziquantel (40 mg/kg) (ShinPoong Pharma., Seoul, Republic of Korea) and a single 400 mg tablet of albendazole (GSK, London, UK), or a half tablet for children aged under two years. On the basis of a positive blood film, or Paracheck© test, non-pregnant women and children were offered Lonart (20 mg/120 mg artemether/lumefrantrine medication; Cipla, Mumbai, India) while pregnant women were offered quinine sulphate tablets (Zest Pharma, Madhya Pradesh, India), as supervised by the project nurse and monitored the following day.

### 2.3 GPS data logger and mapping

A total of 15 GPS-data loggers (I-GotU GT-120, Mobile Action, UK) were available for this study. After completing a brief baseline acceptance survey questionnaire, mothers selected at random were requested to carry this small unit (dimension 44.5 x 28.5 x 13 mm, weight 20 g) back to their homestead, returning it to the field medical team the same day. The unit was powered by a rechargeable 230 mAh Lithium-ion battery which, if set for GPS-data logging at 3-minute intervals, lasts for up to three days before needing recharging. The units were ‘locked’ electronically to avoid any external tampering.

Upon receipt of the unit, data were offloaded onto a personal computer onsite as GPX files which were then used directly in GoogleEarth 5 (Google Inc., CA, USA) and ArcView 9.3 (ESRI, CA, USA) GIS. Using the log it was possible to ascertain, more easily, the position of the homestead. Whilst identity records were kept anonymous, the infection status of each mother and child was used to annotate the maps to reveal any micro-patterning.

To investigate the positional accuracy of the I-GotU device, the lead author accompanied 15 mothers back to their household whilst carrying a Garmin Oregon 550t handheld unit (Garmin, KS, USA). These track logs were later downloaded and directly compared against those obtained from the I-GotU.

### 2.4 Spatial scan statistics

To identify clustering of infection, a spatial scan statistic (Satscan v9.1) was performed.^23^ Based on an expectation of Poisson distribution of cases of infection amongst all possible subject locations surveyed, the spatial scan statistic considered whether the number of cases in an area was excessively high or low. The scan consisted of placing circles of varying radius distances centred at each subject's household location, and computing ratios of observed to expected cases. Both clusters of high and low prevalence were searched for in the scan. The scan statistic was performed separately for schistosomiasis, hookworm, and malaria prevalence. Additionally, a scan was performed for multiple parasite infection, i.e., persons with more than one type of parasite infection.

### 2.5 Ethical approval

This study was approved by the London School of Hygiene & Tropical Medicine and the Ugandan Council for Science and Technology. Each recruited subject was provided a study sensitization leaflet showing information about the GPS device in pictorial format.

## 3 Results

As only sporadic cases of *Ascaris lumbricoides* and *Trichuris trichiura* were found, these were omitted from formal analysis. The general prevalence levels of intestinal schistosomiasis, malaria and hookworm infections in our mothers and child cohort, as well as that within the GPS subset are shown in Table 1.

**Table 1:** Number of people surveyed as well as prevalence levels (and 95% CI in %) of malaria, schistosomiasis and hookworms in the different populations – all families included in the large-scale parasitological survey and the subset included in the GPS tagging study. *N* = number of people investigated; *n* = number of individuals diagnosed positive for the infection

	Mothers			Children		
	*n*	*N*	% (95% CI)	*n*	*N*	% (95% CI)
Total population from Bukoba included in the SIMI survey						
Malaria	36	126	28.6 (20.9–37.3)	214	246	87.0 (82.1–90.9)
Schistosomiasis	34	125	27.2 (19.6–35.9)	19	247	7.7 (4.7–11.8)
Hookworm	75	125	60.0 (50.9–68.7)	55	247	22.3 (17.2–28.0)
Total population from the SIMI survey included in the GPS study						
Malaria	17	63	27.0 (16.6–39.7)	109	123	88.6 (81.6–93.6)
Schistosomiasis	16	63	25.4 (15.3–37.9)	8	122	6.6 (2.9–12.5)
Hookworm	39	63	61.9 (48.8–73.9)	25	122	20.5 (13.7–28.7)

From a general comparison of disease prevalence levels across our total cohort and the GPS subset by Fisher's χ^2^ test, there was no statistical imbalance between prevalence of intestinal schistosomiasis, malaria and hookworm for either mother or child groups. The prevalence of schistosomiasis was consistently ranked lowest across the mother and child cohorts. In children, however, malaria was ubiquitous (>85%), while present in approximately 25% of the mothers, whereas hookworm was more common in mothers (approximately 60%) than in children (approximately 20%).

The plot of the locations of the GPS-tagged households in Bukoba village is shown in Figure 2 and the on-the-ground discordance between the GPS recoded on the I-GotU and Oregon 550t for the 15 compared sampled households was negligible (<7m). It is immediately apparent from these points and the background imagery that there is a concentration of households on the northern side of the village approximately 100–300 m away from the lake which contains some 33 households (52.4% of the GPS-tagged subset).

**Figure 3:**
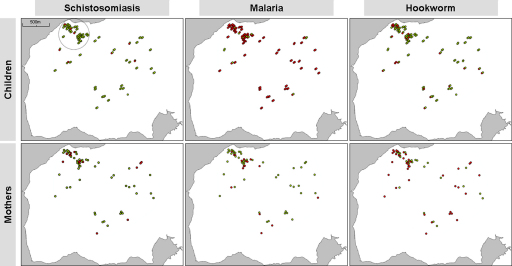
Distribution of schistosomiasis, malaria and hookworms (green for uninfected and red for infected) by household and demographic group (children and mothers). In the top three maps, up to two children (each child symbolised by a circle) may be present per household.

Mapping the distribution of each disease by household for mothers and children is shown in Figure 3. From visual inspection, there was no immediately obvious clustering of infected cases by location, but it is interesting to note households where infection status of mothers and children were either concordant or discordant. General prevalence levels of polyparasitism in our cohort were as follows: triple infection of 1.6% (95% CI 0.2–5.8%) and 4.8% (95% CI 1.0–13.3%) in children and mothers; hookworm and schistosomiasis of 3.3% (95% CI 0.9–8.2%) and 15.9% (95% CI 7.9–27.3%) in children and mothers; hookworm and malaria of 18.0% (95% CI 11.7–26.0%) and 15.9% (95% CI 7.9–27.3%) in children and mothers; and schistosomiasis and malaria 3.3% (95% CI 0.9–8.2%) and 6.3% (95% CI 1.8–15.5%) in children and mothers.

As a simple test for heterogeneity of disease prevalence within the clustered versus non-clustered households, a 500m diameter circle was drawn around this aggregation (see Figure 3) and disease prevalence was calculated inside vs outside (respectively), which for mothers was: malaria 33.3% (95% CI 18.0–51.8%) vs 20.0% (95% CI 7.7–38.6%), intestinal schistosomiasis 33.3% (95% CI 18.0–51.8%) vs 16.7% (95% CI 5.6–34.7%) and hookworm 60.6% (95% CI 42.1–77.1%) vs 63.3% (95% CI 43.9–80.1%); while for children it was: malaria 87.3% (95% CI 76.5–94.4%) vs 90.0% (95% CI 79.5–96.2%), intestinal schistosomiasis 4.8% (95% CI 1.0–13.3%) vs 8.5% (95% CI 2.8–18.7%) and hookworm 20.6% (95% CI 11.5–32.7%) vs 20.3% (95% CI 11.0-32.8%). Again there was no statistical imbalance between prevalence by Fisher's χ^2^ test between inside and outside the circle for either mothers or children, although it is of note that both prevalence of intestinal schistosomiasis and malaria in mothers declined slightly outside this circle.

The results of the scan statistic revealed no significant high or low prevalence clusters for malaria. However, a low prevalence cluster was identified for hookworm (approximately at 0.31°N, 33.5 °E, radius 0.20 km) where there were no cases found in an area expected to have approximately seven cases (*P=*0.072). While we lacked power to detect significant clustering for schistosomiasis, the most likely cluster identified was for a high prevalence region (approximately at 0.31°N, 33.5°E, radius 0.08 km) where there were eight cases in an area expected to have three (*P=*0.81). No significant clustering was found for persons with two or more types of parasite infection.

## 4 Discussion

To our knowledge this is the first report of using GPS-data loggers to record the spatial distribution of households of study participants within a point-prevalence survey. Whilst it is outside the immediate remit of this paper to conduct a detailed multivariate analysis of our data with geospatial models, Figure 2, Figure 3 adequately demonstrate the potential of this methodology to capture the location of each household using small GPS units. Annotating these households by infection status of occupants can very quickly reveal occurrences of disease focality, or proximity to likely infectious sources. The data logging principle has been explored previously using larger units housed inside a wearable waistcoat for mapping the outdoor activities patterns of people tending rice paddies and more recently with I-GotU units for tracking human movements in relation to exposure to infection from dengue viruses.^21^, ^24^

Using the I-GotU to identify the exact position of each household has, in this instance, revealed that the micro-patterning of diseases within Bukoba was not immediately ‘clumped’ which is reassuring that the initial point-prevalence statistic from the 126 households did not contain cryptic micro-patterns, such that, the 63 households that were later geotagged and annotated for each of the three diseases examined were also broadly representative. Of course, this occurrence at Bukoba might not be true of other villages where significant cryptic spatial patterning exists but it is interesting to note that although the differences in disease prevalence were not statistically significant in mothers for malaria and schistosomiasis who lived within the 33 households that were aggregated close to the northern lake shore, there was perhaps the beginnings of a trend for both diseases to decline in the mean prevalence of the other 30 more widely dispersed households. For schistosomiasis it is firmly established that the probability of infection increases with increasing proximity to an infectious water source which might explain this decline as households are located further away from water collection points on the northern shoreline.^19^, ^25^ However, for malaria it could point towards a density dependant effect where transmission is highest amongst people who live in closest proximity to one another.^17^, ^26^

Having the position of the households allows investigations of other factors which might locally perturb the occurrence of infections, as well as control interventions that are set in place to diminish them.^27^ For example, 97.6% of our study cohort reported having recently accessed local health services. Interestingly, 23.6% of mothers reported having to walk for less than one hour with the remaining study population having to walk for 1–4 hours. General access to medications was reasonable and across the village 76% reported having a bednet in their household, with just over half of these reporting it was insecticide treated. We are yet to plot distribution of these bednets across the village but their presence could potentially mitigate the transmission dynamics of anopheline mosquitoes within Bukoba.^27^ For hookworm, only a quarter of our cohort reported regularly wearing sandals, a well-known factor protecting against infection.^6^ It is clear that knowing the location of households enables a new level of geospatial modelling and investigation of risk factors across an examined cohort. As we follow the infection dynamics of these three diseases through time it could also provide new spatial insights into longitudinal processes.^28^ Previous work has shown that in the context of host morbidity knowing the spatial co-occurrence of malaria and schistosomiasis is very important.^13^, ^14^

The on-the-ground accuracy of these units was very good, equivalent to the Oregon 550t unit which is currently tenfold higher in price. However, the Oregon 550t is able to take ‘geostamped’ digital images which is particularly useful as an aide memoire for specific visual points of interest, for example, conditions at each household such as grass thatching or metal roofing, or at points along the way such as small water bodies or agricultural land use.^20^ Most importantly these images can also be uploaded onto GoogleEarth and geospatially aligned to further augment the visual information apparent from the background satellite image. A number of our GPS units malfunctioned in the field some of which resulted in the loss of captured data upon download (eight households). We speculate that the majority of the malfunctions were the result of insufficiently robust hardware for field conditions, often compromising the ability to synchronise with satellites due to humidity and/or water exposure, or due to poor quality software (a common error was the software failing to recognise the device). Nevertheless, the units were well-accepted by the participants and returned in satisfactory condition as seen in other studies.^24^ Given the low cost of the I-GotU 120, approximately £40, a relatively modest financial outlay can lead to exciting possibilities for scaling-up such epidemiological studies to include hundreds of households within a short timeframe. Furthermore, given the widespread use of geospatial referencing in veterinary parasitology, the development of GPS methodology for rapid mapping of human households will allow better integration of data on human and animal parasitic infections and enable potential reservoirs of zoonotic infections to be identified.^29^ Finally, the linkage of infection prevalence data with household locations in a number of villages in different locations could enable identification of common environmental or geographical risk factors associated with particular infections. This could in turn inform control programs so that appropriate measures are implemented at the village, district and national level.

## 5 Conclusions

Using several GPS-devices simultaneously is a rapid and cost-effective way to gather information on the spatial distribution of households during point-prevalence surveys. By revealing cryptic disease micro-patterning, a more detailed insight into local disease epidemiology can be gained.

## Authors’ contributions

JRS conceived the overall rationale for this study set within the Schistosomiasis in Mothers and Infants (SIMI) project conceived by JRS, NBK and JCSF. MB, JCSF and JRS undertook fieldwork and data interpretation. JCSF was responsible for I-GotU devices in the field, entered and analyzed the data. EYWS undertook spatial statistical analysis and participated in general data analysis and interpretation. All authors helped in drafting the manuscript and approved the final version. JRS is guarantor for the paper.

## Funding

The work was supported by a project grant awarded to JRS and NBK from the Wellcome Trust, Gibbs Building, 215 Euston Road, London NW1 2BE, UK.

## Conflicts of interest

None declared.

## Ethical approval

The Ugandan National Council of Science and Technology and the London School of Hygiene & Tropical Medicine, UK, granted ethical approval for these studies (application no. LSHTM 5538·09).
